# Action and rest tremor map to distinct networks within the primary motor cortex

**DOI:** 10.1016/j.celrep.2026.117404

**Published:** 2026-05-28

**Authors:** Lukas L. Goede, Patricia Zvarova, Savir Madan, Bassam Al-Fatly, Xin Xu, Zhipei Ling, Chen Yao, Martin Reich, Jens Volkmann, Calvin Howard, Andrea A. Kühn, Michael D. Fox, Andreas Horn

**Affiliations:** 1 Department of Neurology with Experimental Neurology, Movement Disorders and Neuromodulation Unit, Charité – Universitätsmedizin Berlin, corporate member of Freie Universität Berlin and Humboldt- Universität zu Berlin, Berlin, Germany; 2 Center for Brain Circuit Therapeutics, Department of Neurology, Brigham & Women’s Hospital, Harvard Medical School, Boston, MA, USA; 3 Einstein Center for Neurosciences Berlin, Charité – Universitätsmedizin Berlin, corporate member of Freie Universität Berlin and Humboldt- Universität zu Berlin, Berlin, Germany; 4 Institute for Network Stimulation, Department of Stereotactic and Functional Neurosurgery, University Hospital Cologne, Cologne, Germany; 5 Department of Neurosurgery, Chinese PLA General Hospital, Beijing 100853, China; 6 Department of Neurosurgery, Hainan Hospital of Chinese PLA General Hospital, Sanya, Hainan 572000, China; 7 Department of Neurosurgery, The National Key Clinic Specialty, Shenzhen Key Laboratory of Neurosurgery, the First Affiliated Hospital of Shenzhen University, Shenzhen 518035, China; 8 Department of Neurology, University Hospital Würzburg, Würzburg, Germany; 9 MGH Neurosurgery & Center for Neurotechnology and Neurorecovery (CNTR) at MGH Neurology Massachusetts General Hospital, Harvard Medical School, Boston, MA, USA; 10 Lead contact

## Abstract

Tremor is a common symptom in movement disorders such as Parkinson disease and essential tremor. While both conditions benefit from deep brain stimulation (DBS), the neural substrates underlying different tremor types and their treatment remain poorly defined. Here, we use DBS network mapping in multiple patient cohorts to investigate whether rest vs. action tremor respond to stimulation of the same or distinct subnetworks within the primary motor cortex. Building on recent functional parcellations of the motor cortex, we test whether therapeutic networks converge on either “effector”-specific or “inter-effector” regions along the motor strip. In both disorders and stimulation targets, rest tremor is more strongly linked to effector-specific regions, while action tremor preferentially engages inter-effector territories. Furthermore, clinical programming aligns with symptom-specific network engagement supporting tailored stimulation strategies. These findings provide insights into the network organization underlying tremor types and their treatment, potentially informing symptom-specific neuromodulation strategies across movement disorders.

## In brief

Goede et al. show that different tremor types engage distinct regions of the motor cortex. Using deep brain stimulation network mapping, they find that optimal treatment of rest tremor involves effector-specific areas, while action tremor engages inter-effector regions, supporting personalized stimulation strategies for movement disorders.

## Graphical Abstract

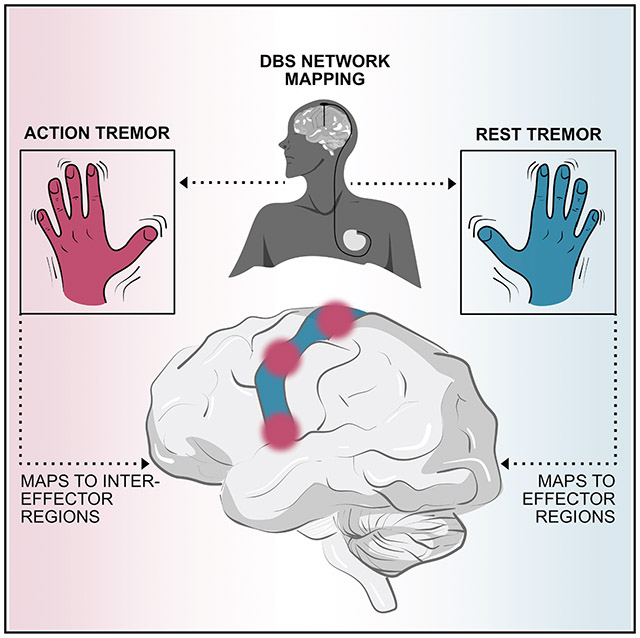

## INTRODUCTION

Tremor is one of the most prevalent motor symptoms in neurological disorders and is a cardinal sign in both essential tremor (ET) and Parkinson disease (PD).^1^ Despite differences in pathophysiology, these disorders share a common challenge: tremor can persist in a subset of patients despite pharmacological treatment and may significantly impair quality of life. Deep brain stimulation (DBS) has proven highly effective in alleviating tremor in both conditions, targeting structures such as the ventral intermediate nucleus (VIM) or subthalamic nucleus (STN).^2,3^

**Figure 1.**
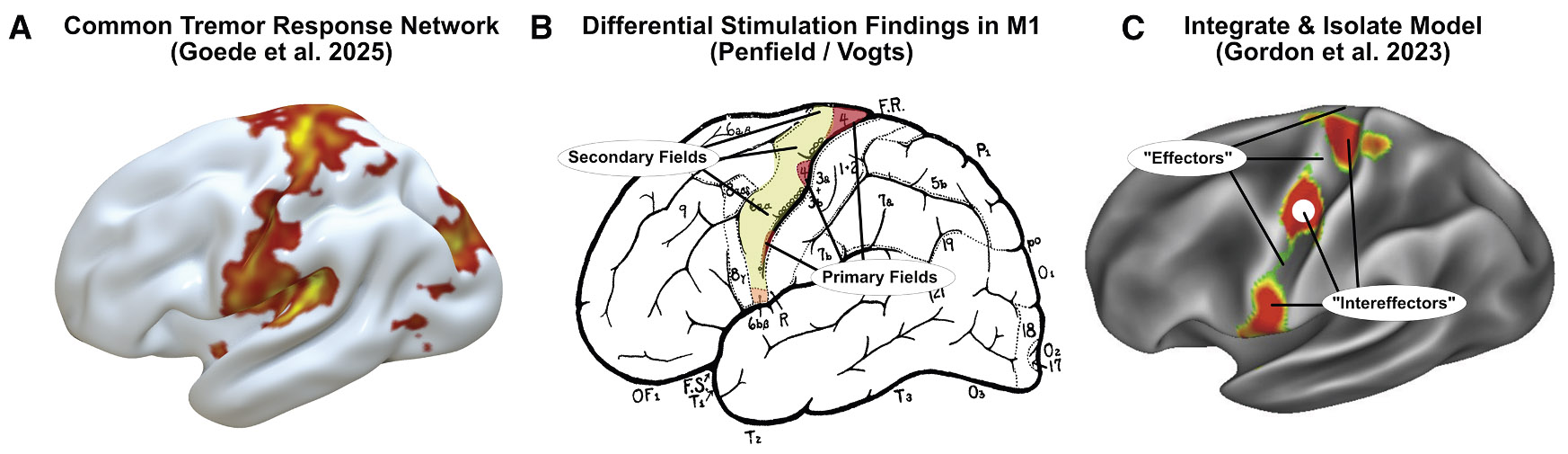
Tremor maps onto primary motor cortex—but precisely onto which of its subregions? (A) Using multimodal convergent mapping techniques, Goede et al. recently described a tremor response network that primarily mapped onto M1, along with cerebellar and thalamic nodes.^4^ Stimulation of this network by DBS led to cessation of tremor in both Parkinson disease and essential tremor. (B) In the first paper that introduced the term “homunculus,” Wilder Penfield reproduced Oskar and Cécile Vogt’s map of differential regions of M1, which required different stimulation thresholds to elicit stimulation responses.^5,6^ (C) A century later, Gordon et al. identified similarly interdigitated subregions of M1 using functional MRI, which they termed “effector” vs. “inter-effector” regions.^8^

A recent study leveraged DBS network mapping, lesion network mapping, atrophy network mapping as well as EMG-fMRI to define a convergent tremor treatment network centered on the cerebellum and the primary motor cortex (M1; Figure 1A).^4^ However, these structures are not homogeneous. Specifically, M1 was recently parcellated into distinct subregions with unique functional connectivity profiles, motivating further investigation of tremor topography within these subdivisions.

The term “homunculus” in the context of the precentral gyrus was introduced by Penfield and Boldrey,^5^ building on earlier work, including direct electrical stimulation experiments by Oskar and Cécile Vogt.^6^ These experiments had combined primate stimulation and human cytoarchitectonic data from the 1920s to delineate three subregions along the motor strip. The “primary fields” (red areas in Figure 1B) required lower stimulation thresholds to elicit motor responses, while the “secondary field” (yellow areas in Figure 1B) needed higher intensities but produced similar motor effects. Additionally, the Vogts described a bipartite ventral area (6b) that, when stimulated, evoked rhythmic movements of the tongue, mouth, pharynx, and larynx (light red in Figure 1B).

About a century later, a similarly interdigitated parcellation of M1 was rediscovered using fMRI-derived resting-state connectivity.^7,8^ In this landmark effort, parts of the “inter-effectors” (likely corresponding to the Vogts’ primary fields) were highly correlated with each other, i.e., they were typically active at the same time as measured by the BOLD signal acquired at rest. In addition, these three “inter-effector” regions connected with subcortical sites such as the centromedian nucleus of the thalamus, dorsolateral putamen, and motor cerebellum and were termed the somato-cognitive action network (SCAN).

The study by Goede et al.^4^ mapped various forms of tremor onto a common network and identified the primary motor cortex as a key cortical node alongside the cerebellum. Here, we wondered whether different forms of tremor would differentially engage subsystems within M1 (secondary fields/effectors vs. primary fields/inter-effectors) and hypothesized that rest tremor would primarily involve effector-specific motor representations, whereas action tremor may recruit inter-effector regions tied to broader action planning and coordination.

Based on a particularly large cohort of DBS patients, here, we tested these hypotheses using DBS network mapping. The analyzed cohort included multiple international DBS cohorts treated for ET or PD, spanning rest and action tremor phenotypes across these two disorders. By mapping stimulation sites to therapeutic networks and analyzing their overlap with distinct subdivisions of M1, we aimed to refine the topography of tremor within motor cortex and explore whether symptom type aligns with the emerging dual-structure model of M1.

## RESULTS

### Clinical cohorts and tremor improvement

We screened data from three DBS cohorts in which full tremor scores with all sub items were available. Specifically, for PD, data were screened from 41 patients treated at Beijing Tiantan Hospital, China, and 97 patients from Würzburg University Hospital, Germany. For ET, 36 patients treated at Charité – Universitätsmedizin Berlin were included. An overview of all cohorts is provided in Table S1. All datasets have been described in detail previously.^9,10^

Rest and action tremor were analyzed as symptom-specific dimensions rather than mutually exclusive patient groups. Hemispheres were included in a given analysis only if the corresponding baseline tremor sub-score was ≥2, ensuring sufficient symptom severity and measurable improvement potential. Consequently, individual patients could contribute hemispheres to rest tremor, action tremor, or both analyses, depending on their baseline symptom profile.

After applying the exclusion criterion of a baseline tremor score, the final analysis included hemisphere-level data as follows: in the subthalamic nucleus (STN) cohort, 42 hemispheres from 30 patients were included for action tremor and 58 hemispheres from 46 patients for rest tremor analysis.

In the ventral intermediate nucleus (VIM) cohort, 62 hemispheres from 34 patients were included for action tremor and 14 hemispheres from 12 patients for rest tremor. A detailed hemisphere-level inclusion flow by geographical cohort and DBS target is provided in Figure S1.

In the STN cohort, the mean baseline rest tremor score on the MDS-UPDRS Part III was 2.57 ± 0.63, and the mean improvement was 1.94 ± 1.02 points, reflecting a 75.5% reduction. For action tremor, the mean baseline score was 2.40 ± 0.54, with a mean improvement of 1.33 ± 0.98 points, corresponding to a 55.4% reduction.

In the VIM cohort, the mean (±SD) baseline Fahn-Tolosa-Marín (FTM) rest tremor score was 2.14 ± 0.36, with a corresponding mean improvement of 2.14 ± 0.36 points, indicating complete (100%) symptom resolution. For action tremor in the same cohort, the mean baseline score was 2.79 ± 0.77, and the mean improvement was 1.79 ± 0.89 points, corresponding to a 64.2% reduction.

### DBS cohort comparison

**Figure 2.**
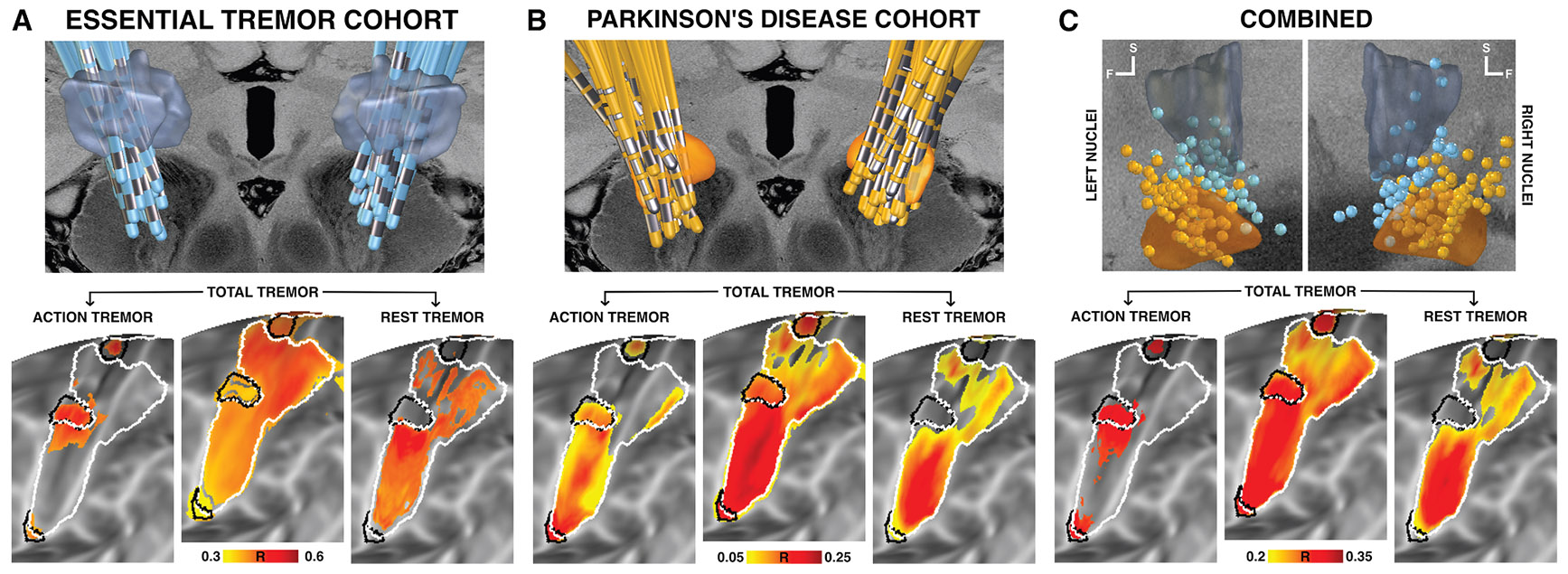
Electrode localizations and tremor-type specific connectivity profiles (A–C) Top row: DBS electrode localizations reconstructed using Lead-DBS^26^ are shown for the essential tremor (ET) cohort targeting the ventral intermediate nucleus (VIM; blue electrodes, A), the Parkinson disease (PD) cohort targeting the subthalamic nucleus (STN; orange electrodes, B), and both cohorts combined in (C), where active contacts are shown as colored spheres. Atlas structures from the DISTAL atlas^27^ are overlaid, with the VIM in blue and the STN in orange. Bottom row: connectivity profiles, restricted to the M1 region, associated with maximal improvement in total tremor, action tremor, and rest tremor are visualized together with the “effector” vs. “inter-effector” M1 subregions defined by Gordon et al.^8^ Across both cohorts, total tremor improvement was associated with connectivity to both regions. However, action tremor preferentially mapped to inter-effector zones (“primary fields” as introduced by the Vogts, black outlines), while rest tremor was more strongly associated with effector-specific regions (Vogts’ “secondary fields,” white outlines). These dissociations were significantly higher than expected by chance across STN (*p* = 0.0474) and VIM (*p* = 0.0348) cohorts as well as when both cohorts were analyzed together (*p* = 0.0472). When repeating the analysis for the effector regions specific for the upper extremities, dissociations were significantly higher than expected by chance across STN (*p* = 0.0268) and VIM (*p* = 0.0164) cohorts as well as when both cohorts were analyzed together (*p* = 0.0212). The observed dissociation statistic exceeded the median of the empirical null distribution in each cohort (STN: 6.59 vs. 0.67; VIM: 10.65 vs. 0.75; combined: 17.24 vs. 1.55; 5,000 permutations), placing the observed values in the extreme tail of the null distribution.

DBS electrode localizations for all cohorts are shown in Figure 2. For each hemisphere, normative functional connectivity profiles of the stimulation volumes were computed and voxel-wise connectivity strength was correlated separately with hemisphere-level improvement in rest and action tremor, yielding symptom-specific correlation maps (R-maps). These maps identify regions whose connectivity to stimulation sites was most strongly associated with improvement in a given tremor type, irrespective of co-occurrence of both tremor types within the same individuals. Replicating previously published findings,^4^ electrodes were associated with greater global tremor improvement when functionally connected to M1 (Figure 2), regardless of stimulation target (STN vs. VIM) and disorder (PD vs. ET). However, action tremor responses were most strongly associated with connectivity to inter-effector regions, whereas rest tremor responses were more strongly associated with connectivity to effector-specific regions within M1. Importantly, this dissociation did not depend on pooling across pathology or stimulation target. The same directional pattern (rest-tremor to effector regions; action-tremor to inter-effector regions) was observed independently within the PD (STN) and ET (VIM) cohorts, indicating that the effect reflects symptom-specific network organization rather than a disorder-specific effect.

Exploratory ROI analysis showed that action tremor inter-effector maps from both ET (VIM) and PD (STN) cohorts exhibited connectivity to the cingulo-opercular network and cerebellar vermis lobule VIIIa (Figure S2).

The association between rest tremor and effector regions vs. action tremor and inter-effector regions was significantly higher than expected by chance based on a permutation analysis in both the STN cohort (see methods, *p* = 0.0474) and the VIM cohort (*p* = 0.0348) or both cohorts combined (*p* = 0.0472). Notably, differences between rest and action tremor were also observed within cerebellar regions across disorders, as illustrated in Figure S3.

To explicitly test whether the effector versus inter-effector dissociation tracks tremor type across disorders, we computed symptom-specific R-maps separately for PD-STN and ET-VIM hemispheres and quantified their relative representation within predefined effector and inter-effector masks. In both cohorts, action tremor showed inter-effector preference, whereas rest tremor showed effector dominance (Figure S4). Importantly, the magnitude of the within-cohort shift from rest to action tremor was comparable across disorders, indicating that the observed network dissociation reflects tremor phenotype rather than diagnosis or stimulation target alone.

Control analyses using normative connectivity derived from the anatomical VIM and STN targets, as well as from cohort-mean active contact locations, did not show differences in effector versus inter-effector connectivity (Figure S5), indicating that the observed pattern cannot be attributed to intrinsic connectivity differences between VIM and STN targets alone but is indeed an effect revealed by the association between clinical improvement and brain connectivity. This suggests that in our cohort, DBS modulates these cortical circuits indirectly, rather than through direct stimulation within the SCAN network.

### Clinician programming reflects symptom-specific network engagement

**Figure 3.**
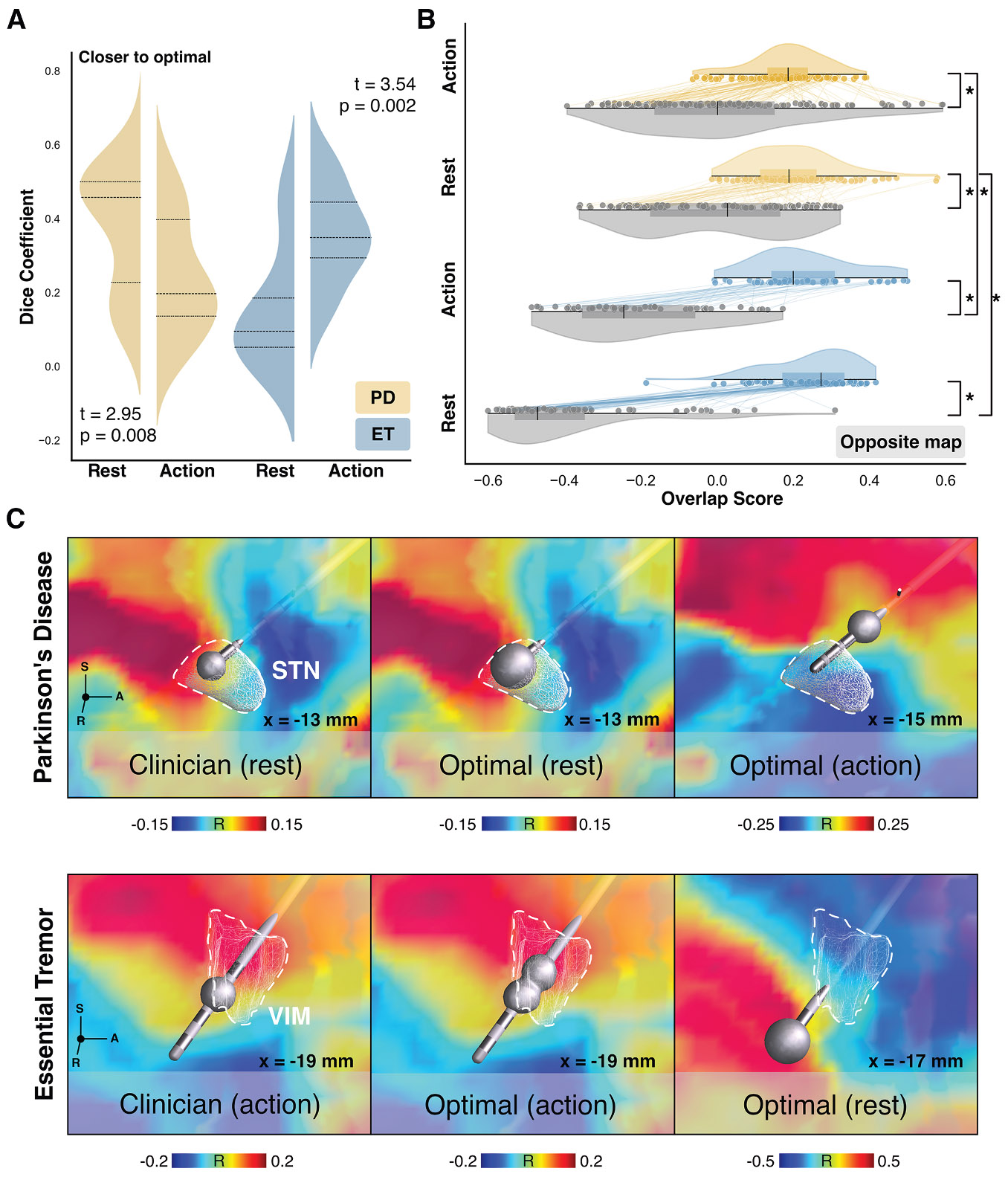
Optimal stimulation volumes can selectively target rest- vs. action-tremor target maps (A) Comparison of the dice coefficient between the stimulation volumes programmed by clinicians and stimulation volumes optimized to match symptom-specific rest- and action-tremor target maps. Clinical stimulation volumes of patients with PD aligned more with rest (left) than action (right) tremor-optimal stimulation volumes (t = 2.95, *p* = 0.008). ET patient clinical stimulation volumes aligned more with action (right) than rest (left) tremor-optimal stimulation volumes (t = 3.54, *p* = 0.002). The three horizontal lines within each violin plot indicate the median (center line) and 25th/75th percentiles (outer lines). (B) Overlap between rest-tremor- and action-tremor-optimized stimulation volumes for PD and ET. While there was no difference in the ability of PD (STN) electrodes to stimulate either network (t = 0.5, *p* = 0.61), ET (VIM) electrodes were able to stimulate the rest tremor network more than the action tremor network (t = 5.2, *p* < 0.0001). Across both diseases, targeting one network compromised stimulation of the other network (t = 19.63, *p* < 0.0001). Embedded box plots indicate the median (vertical center line), interquartile range (box boundaris) and 1.5 times the interquartile range (whiskers) of the overlap scores. (C) Two example reprogramming cases. Top panel: Parkinson disease example with the clinician stimulation volumes (left) and optimal stimulation volumes for the rest (middle) and action (right) tremor networks. Bottom panel: essential tremor example with the clinician stimulation volumes (left) and optimal stimulation volumes for the action (middle) and rest (right) tremor networks.

Above analysis concluded that optimal rest tremor improvement was associated with connectivity to effector regions, while optimal action tremor improvement was associated with connectivity to inter-effector regions. While this was regardless of diagnosis (PD vs. ET; and hence, DBS stimulation target [STN vs. VIM]), it remained unclear whether electrode implantation location itself would preclude from programming in such a way that either network target could well be reached. For each patient, we generated two theoretical stimulation settings that led to stimulation volumes that were maximally connected to either the effector or inter-effector regions. We then compared these stimulation volumes to the ones resulting from the patient’s actual stimulation settings. In the PD cohort, the clinical stimulation volumes were more similar to rest-tremor-focused (effector) than action-tremor-focused (inter-effector) stimulation volumes (t = 2.95, *p* = 0.008). The opposite was seen in ET, where the clinical stimulation volumes were more similar to the action-tremor-focused (inter-effector) than rest-tremor-focused (effector) stimulation volumes (t = 3.54, *p* = 0.002) (Figure 3A).

We next evaluated if both action and rest networks could be equivalently stimulated, regardless of implantation site. STN DBS electrodes engaged both the action and rest tremor networks similarly (t = 0.5, *p* = 0.61), whereas VIM DBS electrodes preferentially stimulated the rest tremor network over the action tremor network (t = 5.2, *p* < 0.0001) (Figure 3B). Regardless, as shown in Figure 3B, for most patients, stimulation settings can be adjusted to substantially engage either network. The VIM DBS electrodes could stimulate the rest tremor network more than the STN DBS electrodes (t = 2.68, *p* = 0.008), although there was a less significant difference between either site’s ability to stimulate the action tremor network (t = 2.54, *p* = 0.01). Interestingly, electrodes programmed to stimulate one tremor network had significantly compromised stimulation of the other network (t = 19.63, *p* < 0.0001) (Figure 3B). To visualize this tradeoff, we plotted the rest- and action-optimal stimulation volumes for one patient with PD (Figure 3C, top) and one patient with ET (Figure 3C, bottom).

## DISCUSSION

This study aimed to disentangle the cortical substrates of different tremor types by leveraging functional subdivisions within the primary motor cortex (M1). Using data from multiple international DBS cohorts across PD and ET, we applied DBS network mapping to investigate whether distinct rest vs. action tremor would differentiate across anatomically and functionally defined subregions of M1. Indeed, rest and action tremor preferentially mapped to effector-specific versus inter-effector zones, respectively (corresponding to secondary and primary fields as defined by the Vogts, respectively). This dissociation could be seen when analyzing the PD STN-DBS cohort and the ET VIM-DBS cohort separately, as well as when pooling across targets.

It is a common belief in our field that Penfield’s motor homunculus^5^—as the most salient model of M1 somatotopy—had suggested a smooth transition without disruptions.^8^ Interestingly, Penfield never made any such claims (or presented any evidence) about a strict linear organization of the motor strip without disruptions. His work built upon earlier contributions from his mentor Otfrid Foerster^11^ and other pioneers such as Fritsch, Hitzig, the Vogts and Sherrington, who had already segregated M1 into functional zones decades before. It may have been the salient model of a “little man” that has seemed suggestive to readers that it made claims about a motor cortex without disruptions. Indeed, earlier anatomical work by pioneers such as Louis Pierre Gratiolet and Paul Broca had suggested disruptions in the form of sulcal continua or “plis de passage” that disrupt M1 homogeneity.^12^ Oskar and Cécile Vogt’s work in collaboration with Otfrid Foerster from 1918 onward had also identified subregions within M1 (Figure 1B) that showed different stimulation thresholds and cytoarchitectonic properties. Much more recently, Germann et al. proposed a novel “homuncular” model that had numerous clearly defined gaps and five distinct regions using morphological analysis and fMRI.^13^ A few years later, a landmark study leveraged resting-state fMRI with extensive methodological effort to reveal segregated regions within M1. In this work, the authors introduced novel terminology and described classical “homuncular” regions as effectors (likely corresponding to Vogts’ secondary fields). In between these effectors, three “inter-effector” regions (likely corresponding to Vogts’ primary fields and sulcal continua or Broca’s plis de passage^12^) formed a functionally connected somato-cognitive action network (SCAN).^8,14^ This novel segregation of M1 networks has led to multiple DBS mapping studies in PD,^14^ Tourette syndrome^15^ and dystonia,^16^ each time asking the question of whether DBS effects would associate with modulating one of these specific M1 subnetworks.

Our results suggest that tremor relief through DBS differentially engages these two systems for different forms of tremor. Specifically, rest tremor, typically predominant in PD, mapped more strongly to effector-specific regions. In contrast, action tremor, characteristic of ET, mapped to inter-effector regions, which are functionally connected to the CON and cerebellar vermis (notably lobule VIIIa), and are thought to support whole-body action planning and control.^8,14^ Critically, in both disorders, the respective other form of tremor can also be present and conclusively mapped to the respective other network. In an exploratory ROI analysis (Figure S2), the action tremor inter-effector maps from both cohorts showed connectivity to the cingulo-opercular network and cerebellar vermis lobule VIIIa. Notably, connectivity in PD was numerically more pronounced, which may reflect broader network recruitment in PD, where tremor coexists with additional motor and non-motor features that engage cerebello-opercular control systems.

Importantly, our finding that rest tremor preferentially maps to effector regions should not be interpreted as reduced cerebellar involvement. Effector territories correspond to classical primary motor cortex representations that are robustly coupled to cerebellar motor regions, as shown in large-scale cerebello-cortical connectivity mapping.^17^ In contrast, inter-effector regions form the cortical backbone of the SCAN, which exhibits prominent integration with distributed cortical control networks and cerebellar nodes.^8,14^ Rather than distinguishing cerebellar versus non-cerebellar systems, our results suggest that rest and action tremor may engage different cortical entry points into cerebello-thalamo-cortical loops, with effector regions participating through more classical somatomotor pathways and inter-effector regions via SCAN-linked circuits. Direct mechanistic comparisons beyond connectomic approaches remain limited and warrant future investigation.

Our findings should be interpreted in the context of recent work by Ren et al. (2026), who conceptualize PD as involving the SCAN. Notably, their analyses relate SCAN connectivity to total MDS-UPDRS-III motor scores, which are predominantly driven by hypokinetic and axial features rather than tremor. In their repetitive transcranial magnetic stimulation (rTMS) experiments, SCAN-targeted stimulation improved bradykinesia and rigidity but did not significantly modulate tremor. In contrast, our study isolates tremor sub-scores and dissociates rest and action tremor at the hemisphere level. Thus, rather than contradicting the SCAN framework, our findings suggest that tremor engages partially distinct motor-cerebellar subnetworks within M1, whereas SCAN connectivity may be more closely linked to hypokinetic motor features.

These results also intersect with advances in precision functional mapping of motor cortex organization. Integrating individualized resting-state functional connectivity mapping with the symptom-specific framework presented here could enable patient-tailored neuromodulation strategies. Specifically, individualized delineation of effector and inter-effector subnetworks may help determine which cortical circuit is most relevant for a given patient’s dominant tremor phenotype, potentially refining DBS programming or guiding targeted non-invasive stimulation.

This network specificity is not merely an anatomical distinction, it carries functional and evolutionary implications. The effector-specific system has been hypothesized to align with the corticomotoneuronal (CM) system, which developed as late as in primates and allows for direct cortical control over distal musculature and skilled movements.^18^ In contrast, the inter-effector system, which encompasses the SCAN, cerebellar vermis, and CON, may be phylogenetically older, with homologs in early mammals. The system supports integrative functions including postural regulation, action planning, and physiological coordination.^14,19,20^ Our findings suggest that tremor phenotypes may tap into these evolutionarily distinct motor subsystems, one optimized for fine motor output and the other for broader integrative control. However, direct links between phylogenetic developments and these network structures have not been demonstrated by solid evidence, and these lines of reasoning should be interpreted with caution.

Non-invasive neuromodulation studies using transcranial magnetic stimulation (TMS) have variably targeted M1 or the cerebellum in attempts to alleviate tremor in both PD and ET.^21,22^ However, to our knowledge, TMS studies have not differentiated stimulation targets within M1 based on tremor type, and optimal cortical sites for distinguishing rest from action tremor remain unclear. Our findings may help guide TMS protocols by identifying symptom-specific cortical targets, potentially improving the precision of non-invasive approaches when a particular tremor phenotype predominates.

Finally, these findings may help contextualize why DBS outcomes can vary across individuals, even when lead placement appears accurate by conventional standards. A mismatch between the tremor phenotype (e.g., action vs. rest tremor) and the cortical subnetwork most effectively modulated by a given DBS target could underlie some cases of suboptimal response. For instance, targeting cerebello-thalamic projections that primarily modulate SCAN-linked cortical areas might benefit action tremor more than rest tremor, which appears to be more closely tied to effector-specific M1. Beyond its implications for target selection, our findings also highlight the potential for revised programming strategies that are tailored to symptom-specific tremor networks. We observed that the implanted electrodes could, in most cases, be reprogrammed to modulate specific cortical subsystems to better align with a patient’s tremor phenotype. This approach could enhance personalization of DBS therapy and improve outcomes in patients with mixed tremor symptoms.

### Limitations of the study

Several limitations should be considered when interpreting our findings. First, although we pooled data across multiple international cohorts to increase generalizability, patient-level heterogeneity, such as differences in disease severity, treatment history, imaging quality, and clinical scales, may limit comparability across sites. A limitation inherent to multi-center neuroimaging studies is potential variability in brain morphology across populations, age groups, and scanner platforms. Population-specific brain templates, such as those described by Liang et al.,^23^ have been shown to reduce deformation during spatial normalization in certain cohorts. In the present study, all data were normalized to MNI space using diffeomorphic registration with additional local refinement (WarpDrive^24^) to optimize subcortical alignment and electrode localization. Given that DBS network mapping primarily depends on accurate mesoscopic localization of stimulation relative to subcortical targets, fine-grained cortical morphometric differences are less likely to systematically bias the core analyses. Nonetheless, we acknowledge the use of a common MNI template across ethnically diverse cohorts as a limitation and encourage future work to evaluate population-specific atlas frameworks where available. Also, the use of a normative connectome rather than patient-specific functional imaging may be a limitation. While this increases stability and signal-to-noise of connectivity estimates, it does not capture disease-specific or individual variability in network organization. Additionally, examining a cohort consisting exclusively of patients with tremor-dominant PD would be valuable to determine whether similar patterns hold within this more homogeneous subgroup. In particular, the relatively small VIM/rest subgroup limits statistical power and may reduce the stability of effect estimates in that comparison, warranting cautious interpretation. A further limitation of the present study is the restricted dynamic range inherent to clinically used tremor rating scales. Hemispheres were included only if baseline tremor severity was ≥2, and tremor subitems on both the MDS-UPDRS-III and Fahn-Tolosa-Marin scale are ordinal measures with limited granularity. Although improvement scores in our dataset spanned the full possible range (0–4 points) and Spearman correlations are rank-based and therefore robust to non-normal distributions and restricted scaling, the use of ordinal clinical ratings may limit sensitivity compared with continuous quantitative tremor measures. Future studies integrating objective accelerometry or kinematic recordings may enable higher-resolution symptom-connectivity mapping. Further, the analyses are correlational in nature. Although DBS provides a causal framework by linking stimulation to symptom change, prospective studies and lesion-based approaches are needed to further validate causal relationships.^25^ Finally, sex- and gender-based analyses were not performed due to limited sample size and incomplete demographic data across cohorts, which may restrict the generalizability of our findings.

In summary, our results segregate the functional topography of neuromodulatory treatment of tremor within the primary motor cortex. By mapping tremor-type-specific therapeutic networks onto M1 subdivisions, we demonstrate that action and rest tremor engage functionally and evolutionarily distinct cortical motor systems. These findings offer a step toward personalized neuromodulation strategies and provide a testable framework for understanding variability in tremor pathophysiology and DBS treatment outcomes. Future studies should assess whether this symptom-network mapping generalizes to other disorders and stimulation modalities, including noninvasive approaches, and whether aligning therapeutic targets with symptom-specific cortical networks can enhance clinical efficacy.

## STAR★METHODS

### EXPERIMENTAL MODEL AND STUDY PARTICIPANT DETAILS

#### Human study participants

All procedures were conducted in accordance with the Declaration of Helsinki and approved by the institutional review board of Brigham and Women’s Hospital (master vote 2020P002987). We retrospectively included patients with Parkinson’s disease (PD) or essential tremor (ET) who had undergone deep brain stimulation (DBS). Data of patients with PD were derived from previously published cohorts that underwent surgery at DBS centers in Würzburg and Beijing,^9,28^ while the ET cohort consisted of patients operated in Berlin.^10^ Patients with baseline tremor scores below two per tremor type were excluded to ensure sufficient symptom severity for reliable assessment of clinical improvement, as done previously^9^ (patients with almost no tremor at baseline cannot truly improve, i.e., are not informative for this type of analysis). A summary of all patient cohorts is provided in Table S1.

### METHOD DETAILS

#### Clinical tremor assessment

Tremor severity was assessed using validated, disorder-specific clinical scales. For ET, we used the Fahn-Tolosa-Marin (FTM^29^) Tremor Rating Scale, while for PD, we used the Movement Disorder Society Unified Parkinson’s Disease Rating Scale, Part III (MDS-UPDRS Part III).^30^ For each hand, subitems measuring rest- and action tremor were scored on a 5-point scale (0–4). Given the often lateralized nature of tremor symptoms, clinical scores were analyzed as hemisphere-specific (hemi-scores) and assigned to the anatomically contralateral DBS electrode, consistent with prior work.^4,10^

#### Electrode localization and stimulation volume modeling

DBS electrodes were localized using default pipelines in Lead-DBS software (version 3).^26^ Briefly, preoperative MRI and postoperative CT or MRI scans were linearly co-registered using advanced normalization tools (Advanced Normalization Tools; ANTs, http://stnava.github.io/ANTs/). To correct for intraoperative brain shift due to pneumocephalus, Lead-DBS’s integrated correction was applied. Multi-spectral preoperative volumes were then used to compute a spatial normalization warp field into ICBM 2009b Nonlinear asymmetric (‘MNI’) space using the SyN Diffeomorphic Mapping approach implemented in ANTs.^31^ Normalization results were checked and in cases where normalization errors were clearly visible and image quality allowed refinement, the integrated toolbox WarpDrive was used to reach optimal normalization results for subcortical structures.^24^ Subsequently, electrodes were reconstructed using the PaCER algorithm for CT-based imaging or the TRAC/CORE method for MRI-based imaging, followed by manual inspection and refinement. Anatomical segmentations of subcortical structures were defined using the DISTAL atlas, integrated within the Lead Group analysis tool.^27^ Estimation of the volume of tissue activated (VTA) was performed using the OSS-DBS v2 toolbox.^32^

#### Deep brain stimulation network mapping

Functional connectivity profiles (i.e., seed maps) were computed for each VTA using the Lead Connectome Mapper tool,^28^ after non-linearly flipping right-sided stimulation volumes to the left hemisphere for group-level analysis. Seed maps represent average voxelwise functional connectivity between each VTA and the rest of the brain, using a normative resting-state connectome, that was built based on resting state functional connectivity data from 1,000 healthy subjects using a 3T Siemens (Erlangen, Germany) MRI, as part of the Brain Genomics Superstruct Project.^33^

To identify optimal therapeutic networks, we correlated seedmap connectivity values with clinical hemi-score improvements across each cohort (ET-VIM and PD-STN), yielding correlation maps (*R-maps*) of optimal connectivity. Positive R-values indicate regions preferentially connected to electrodes associated with better clinical response, and vice versa. The regions-of-interest maps of the effector- and inter-effector regions were kindly provided as a NifTI file by the group that originally identified these networks.^8^

#### Optimizing deep brain stimulation to fMRI network maps

To determine whether tremor-specific map engagement was primarily driven by clinician programming versus lead placement, we generated two theoretical stimulation settings per patient designed to maximally engage either the action- or rest-tremor map. Optimization was performed using the publicly available StimPyPer algorithm (https://github.com/Calvinwhow/StimPyPer). Rather than relying on computationally intensive biophysical field simulations, this approach uses a fast geometric approximation of stimulation volumes based on contact coordinates and applied current, enabling efficient iterative search.

Candidate programs were evaluated with an objective function that increases the average (or density) of symptom-map target values within the stimulation volume while penalizing excessive total current and clinically implausible amplitudes. To address the non-convex optimization problem, the algorithm initializes current allocation using proximity-weighted contact scoring and then applies gradient-based updates to identify the multi-contact configuration that best targets the respective symptom map.

### QUANTIFICATION AND STATISTICAL ANALYSIS

For correlation analyses, Spearman’s rank correlation coefficients were calculated throughout.

To test whether rest and action tremor differentially engage effector versus inter-effector subregions of M1, inference was performed at the ROI level rather than at the voxel level. Voxelwise R-maps were first generated as Spearman correlation maps relating connectivity to clinical improvement. These maps were then summarized within anatomically defined effector and inter-effector masks by computing the mean correlation value within each ROI, yielding an effector-to-inter-effector ratio for each tremor type and cohort. Our primary statistic was the ratio of rest versus action tremor engagement, where values > 1 indicate relatively stronger effector representation for rest tremor and <1 the opposite.

Statistical significance was assessed using a nonparametric permutation framework (5,000 iterations). At each iteration, clinical improvement scores were randomly shuffled within tremor type and cohort, R-maps were recomputed, and ROI summary statistics recalculated. The observed statistic was then compared against the resulting empirical null distribution. Because inference was performed on ROI-aggregated summary measures rather than voxelwise tests, and because the permutation framework preserves ROI size and spatial structure at each iteration, no voxelwise multiple-comparison correction was required. Spatial similarity between the optimized stimulation volumes and each patient’s clinically active stimulation volume was quantified using Dice coefficients.

## Supplementary Material

1

Supplemental information can be found online at https://doi.org/10.1016/j.celrep.2026.117404.

## Figures and Tables

**Table:** KEY RESOURCES TABLE

REAGENT or RESOURCE	SOURCE	IDENTIFIER
Deposited data		
Normative resting state connectome	Yeo et al.^33^	N/A
Effector-/Inter-effector ROI maps	Gordon et al.^8^	N/A
Software and algorithms		
Lead-DBS (Version 3.0)	Neudorfer et al.^26^	www.lead-dbs.org
Advanced Normalization Tools	Avants et al.^31^	http://stnava.github.io/ANTs/
OSS-DBS toolbox (v2)	Butenko et al.^32^	N/A
StimPyPer algorithm	This paper, C.H.	https://github.com/Calvinwhow/StimPyPer
Other		
DISTAL Atlas	Ewert et al.^27^	www.lead-dbs.org

## Highlights

- Optimal rest and action tremor response maps to distinct M1 subregions
- Rest tremor associates with effector-specific M1 regions in PD and ET
- Action tremor preferentially engages inter-effector regions linked to SCAN in PD and ET
- DBS electrodes can be reprogrammed to selectively target tremor-specific networks Goede et al., 2026, Cell Reports *45*, 117404
