# Supplementary material for: Action and rest tremor map to distinct networks within the primary motor cortex
Source: Cell Rep. Author manuscript; Available in PMC 2026 Jul 20. (PMC13382931; doi:10.1016/j.celrep.2026.117404)
Supplement: 1 [file NIHMS2190703-supplement-1.pdf]

Cell Reports, Volume *45*

Supplemental information

Action and rest tremor map to distinct networks within the primary motor cortex

Lukas L. Goede, Patricia Zvarova, Savir Madan, Bassam Al-Fatly, Xin Xu, Zhipei Ling, Chen Yao, Martin Reich, Jens Volkmann, Calvin Howard, Andrea A. Kühn, Michael D. Fox, and Andreas Horn

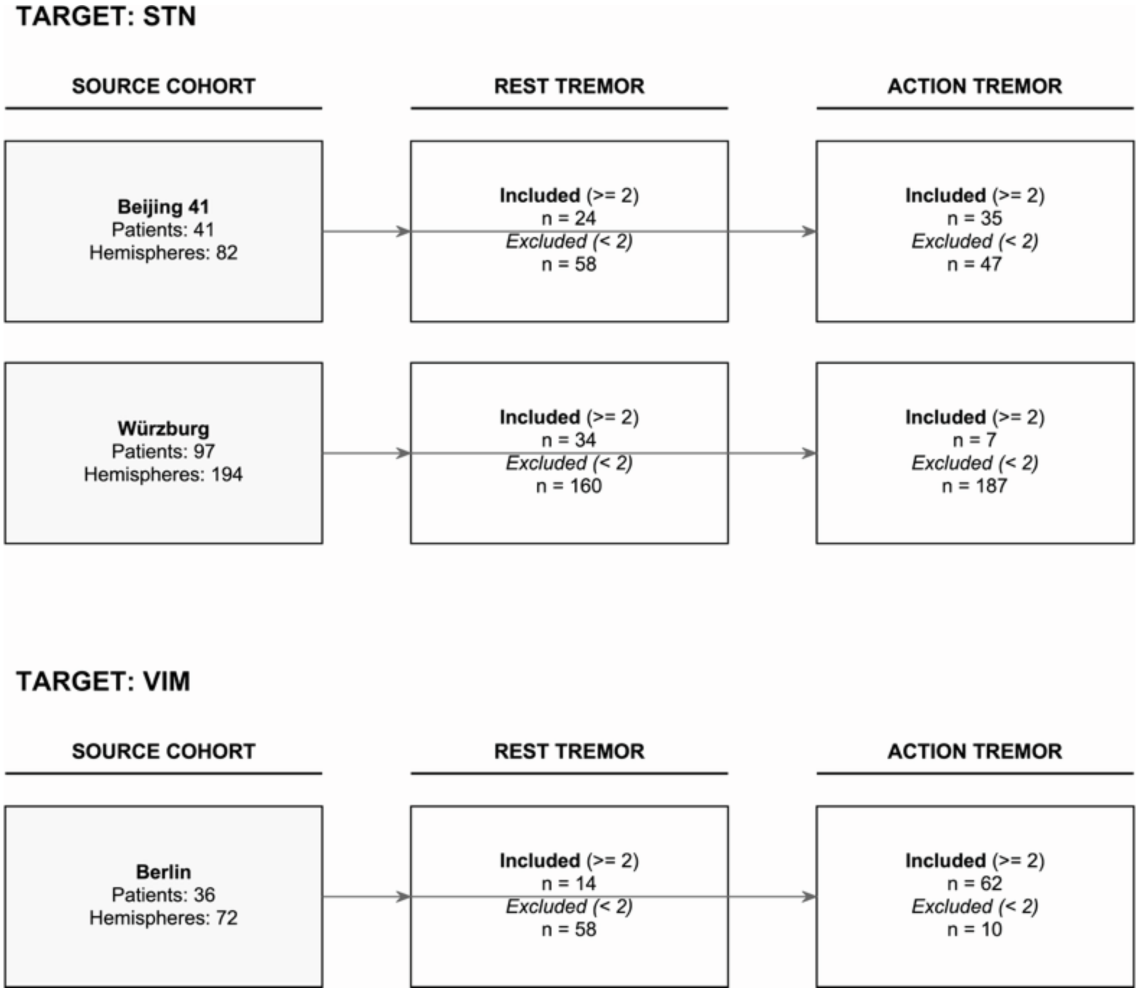

Supplementary Figure S1. Cohort inclusion flow. Hemisphere-level inclusion flow for STN and VIM DBS cohorts by geographical source. Rest and action tremor were analyzed separately; hemispheres were included only if the respective baseline tremor sub-score was ≥2. Numbers denote hemispheres.

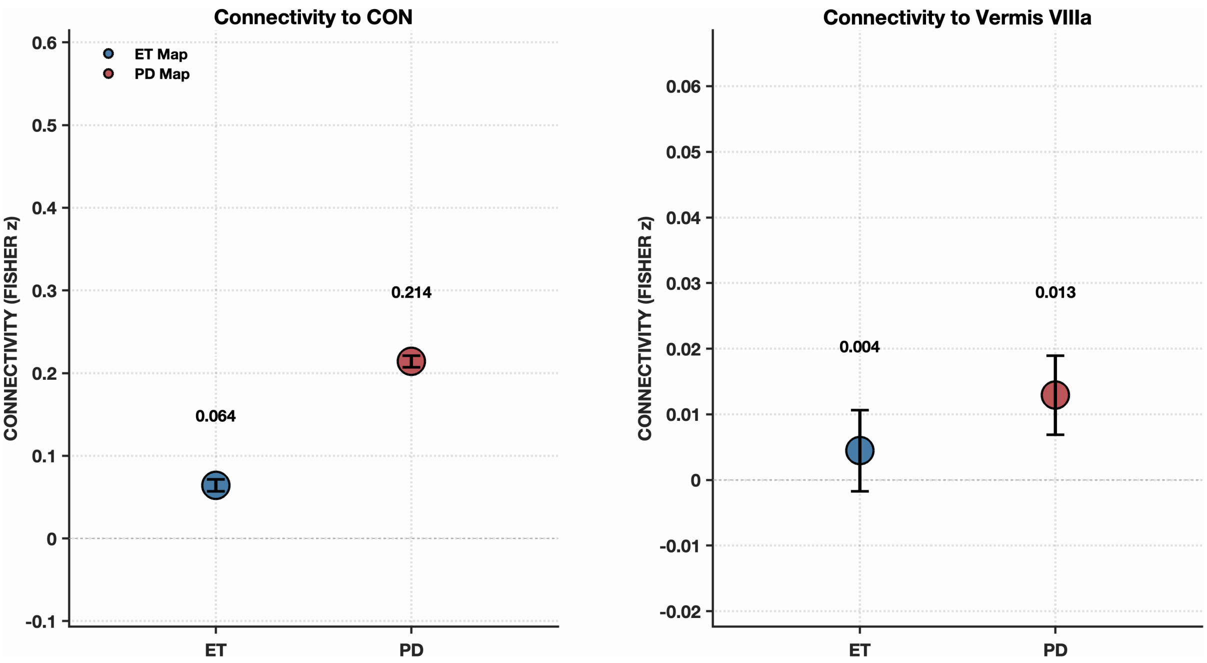

Supplementary Figure S2. ROI connectivity of action tremor inter-effector maps. Scatter plots show Fisher z-transformed connectivity values between action tremor inter-effector R-maps from the ET/VIM and PD/STN cohorts and two regions of interest: the cingulo-opercular network (CON; left) and cerebellar vermis lobule VIIIa (right). Vermis VIIIa corresponds to label 18 of the Diedrichsen probabilistic cerebellar atlas (SUIT space) and was exported and resliced to MNI space for analysis. Error bars represent ±1 Standard Error of the Mean (SEM).

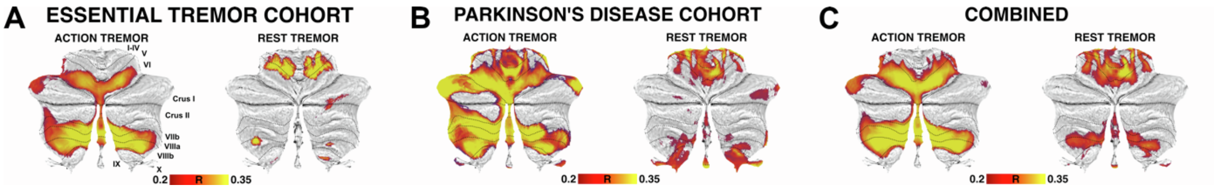

Supplementary Figure S3. Tremor-type specific connectivity profiles within the cerebellum. Connectivity profiles reveal distinct cerebellar patterns when comparing action versus rest tremor across disorders: essential tremor (A) and Parkinson's disease (B). Roman numerals in panel (A) indicate cerebellar lobules. Panel (C) shows combined cohort maps. Cerebellar flatmaps were created using the SUIT toolbox.

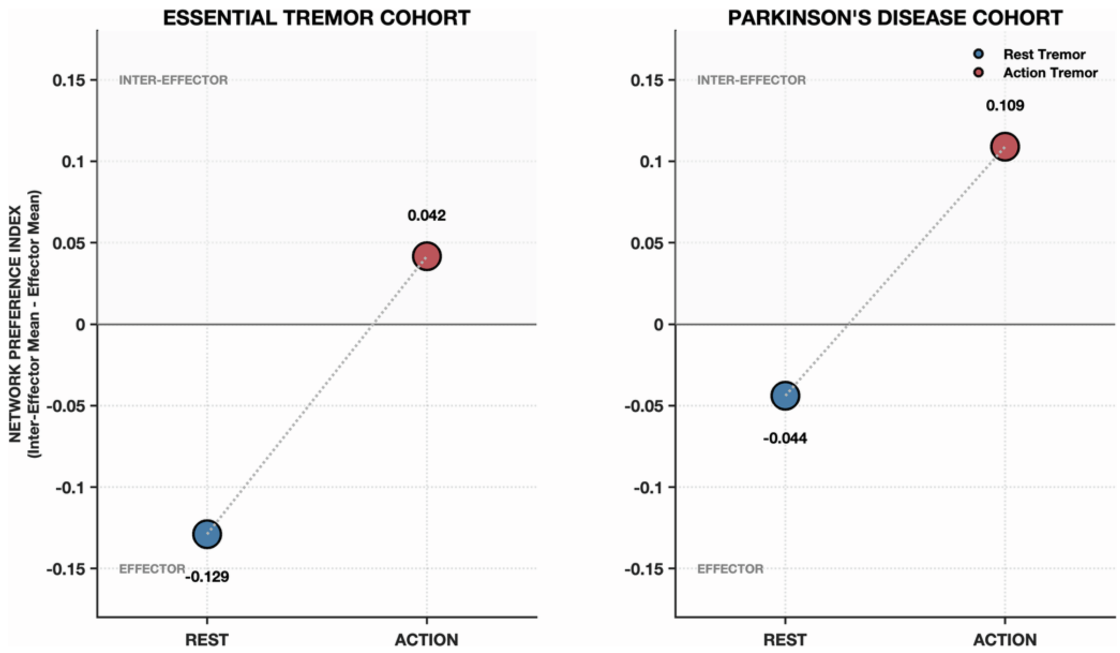

Supplementary Figure S4. Effector versus inter-effector engagement in symptom-specific R-maps across disorders. Quantification of effector versus inter-effector representation in symptom-specific R-maps computed separately for PD-STN and ET-VIM cohorts. The network preference index is defined as the mean R-value within the inter-effector mask minus the mean R-value within the effector mask (Inter-effector minus Effector). Positive values indicate relative inter-effector emphasis, whereas negative values indicate effector dominance. In both cohorts, action tremor shows inter-effector preference, while rest tremor shows effector dominance.

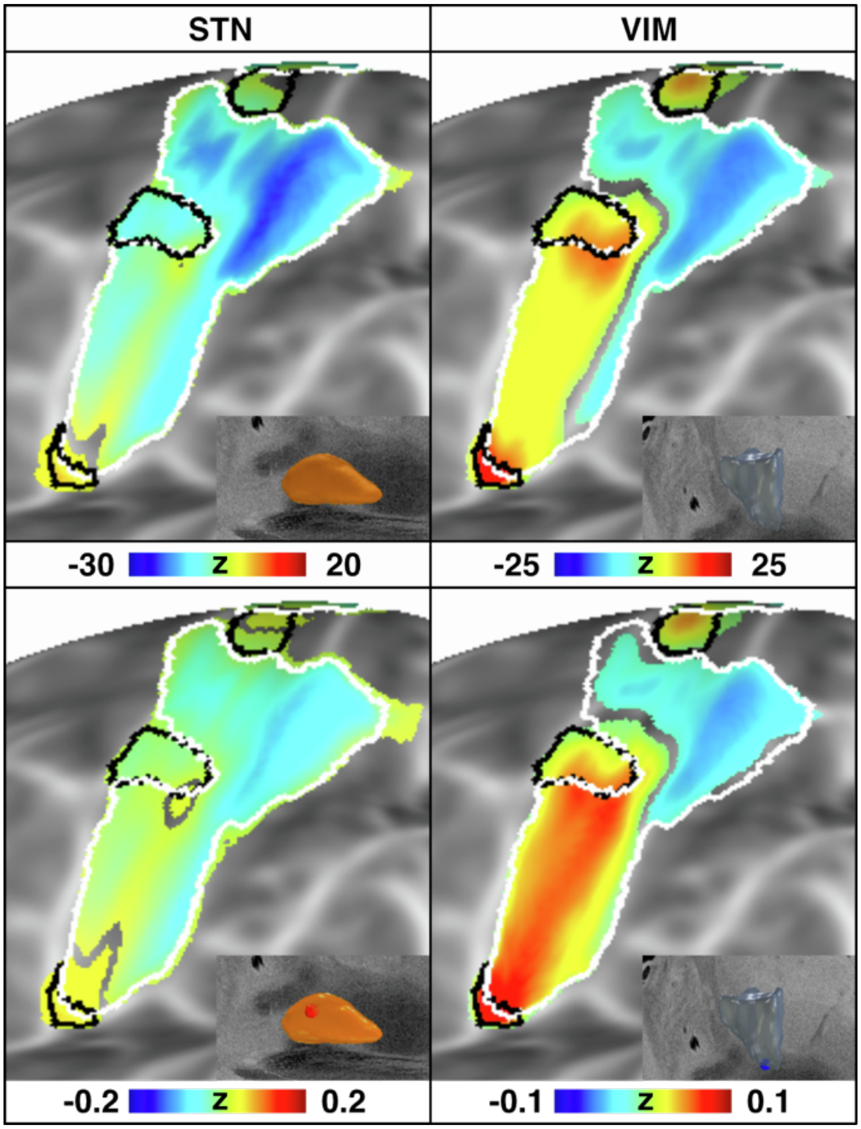

Supplementary Figure S5. Target-level and cohort-mean connectivity profiles for VIM and STN DBS. Top row: Normative functional connectivity of the anatomical target structures, using the subthalamic nucleus (STN; left) and the ventral intermediate nucleus of the thalamus (VIM; right), defined by the DISTAL atlas as seeds. These maps reflect target-level connectivity independent of patient-specific stimulation settings.

Bottom row: Normative functional connectivity derived from the cohort-mean active contact location for each DBS cohort. Mean active contacts were computed across patients and hemispheres after mirroring to a common hemisphere. All maps are shown as Fisher z-transformed connectivity.

Supplementary Table S1. Information about the patient cohorts.

**Center**	Beijing, China	Würzburg, Germany	Berlin, Germany
	**Demographic information**		
**Surgical DBS Center**	Chinese PLA General Hospital	University Hospital Würzburg	Department of Neurology, Charité – Universitätsmedizin Berlin
**N (female)**	41 (21)	97 (31)	36 (14)
**Age at time of surgery (mean ± SD; in years)**	61 ± 10	60 ± 8	74 ± 12
	**Clinical Details**		
**Time of follow-up (in months)**	N/A	N/A	12 ± 9.86
**Surgical target**	STN	STN	VIM
**Score used to assess tremor**	MDS-UPDRS-III	MDS-UPDRS-III	FTM
**Tremorscore (rest+action) at baseline (mean ± SD)**	2.04 ± 1.83	0.82 ± 1.29	3.14 ± 1.41
**Tremorscore (rest+action) at time of follow-up under stimulation ON condition (mean ± SD)**	1.13 ± 1.35	0.21 ± 0.62	1.00 ± 0.87
**Relative improvement (mean ± SD; in %)**	48.2 ± 46.0	74.0 ± 63.4	63.3 ± 37.7
**Absolute improvement (mean ± SD)**	0.90 ± 1.47	0.60 ± 1.24	2.14 ± 1.54
	**Imaging and DBS Specification**		
**Imaging modality (post-operatively)**	CT	CT	CT (N=24) / MRI (N=12)
**Electrode models**	Medtronic 3389	Boston Scientific Vercise Directed/Boston Scientific Vercise	Medtronic 3387/Boston Scientific Vercise Directed/St. Jude ActiveTip (6142-6145)
**Related citation**	Rajamani et al.	Rajamani et al.	Al-Fatly et al.

Abbreviations: DBS, Deep brain stimulation; SD, standard deviation; FTM, Fahn-Tolosa-Marin Tremor Rating Scale; STN, Subthalamic nucleus; MDS-UPDRS-III, Movement Disorder Society Unified Parkinson’s Disease Rating Scale, part III; VIM, Ventral intermediate nucleus of the thalamus.
